# Dysglycemia With Impaired Insulin Secretion After Resection of a High-Molecular-Weight IGF-II–Producing Tumor

**DOI:** 10.1210/jcemcr/luac013

**Published:** 2022-11-29

**Authors:** Yasuho Shimada, Koh Yamashita, Izumi Fukuda, Toru Aizawa

**Affiliations:** Division of Internal Medicine, Department of Diabetes, Endocrinology and Metabolism, Shinshu University School of Medicine, Matsumoto 390-8621, Japan; Diabetes Center, Aizawa Hospital, Matsumoto 390-8510, Japan; Department of Endocrinology, Metabolism and Nephrology, Graduate School of Medicine, Nippon Medical School, Tokyo 113-8603, Japan; Diabetes Center, Aizawa Hospital, Matsumoto 390-8510, Japan

**Keywords:** high-molecular-weight insulin-like growth factor-II, β-cell function, insulin

## Abstract

Analysis of insulin and related glucoregulatory hormone secretion following high-molecular-weight insulin-like growth factor II (HMW-IGF-II)–releasing tumor excision has never been reported. In a man with chronic hypoglycemia—plasma glucose (PG), 2.1 mmol/L with undetectable serum insulin, less than 7.2 pmol/L on admission—the cause of the hypoglycemia was HMW-IGF-II in the serum secreted by an intrathoracic benign pleural solitary fibrous tumor (size: 15 × 17 × 12 cm). Removal of the tumor nullified serum HMW-IGF-II and hypoglycemia. Postoperative glucose metabolism was evaluated day 272 by 75 g oral glucose tolerance test (OGTT) and on days 5, 202, and 990 by fasted sampling. Glycated hemoglobin A_1c_ (HbA_1c_) was 37 to 41 mmol/mol, fasting PG was 5.3 to 5.4 mmol/L, and 2-hour PG at 75 g OGTT was 6.9 mmol/L, indicating that he was at the prediabetes stage. Homeostasis Model Assessment 2 of Insulin Resistance and Homeostasis Model Assessment 2 of β-Cell levels were within the normal range but the Stumvoll first phase was lowered. Insulin sensitivity and secretion were compared to age-, sex-, and body mass index–matched controls with normal glucose metabolism. Long-term HMW-IGF-II exposure of pancreatic islet β cells caused the functional impairment, that is, suppressed glucose-stimulated insulin secretion (GSIS), leading to nondiabetic hyperglycemia. This fact suggests long-term HMW-IGF-II exposure of the islet β cell specifically dampens GSIS.

Non–islet cell tumor hypoglycemia (NICTH) was first reported by Nadler and Wolfer in 1929, and a causal role of tumor produced insulin-like growth factor II (IGF-II), especially the high-molecular-weight (HMW) variant, was demonstrated in NICTH by Daughaday et al in 1988. Since then, HMW-IGF-II–induced NICTH has been widely reported in epithelial and mesenchymal tumors as well as hepatocellular carcinoma, and concurrent NICTH and serum HMW-IGF-II have been observed in patient serum [1]. Thus, patients with NICTH having HMW-IGF-II identified in the serum per se are not uncommon. Surprisingly, analysis of insulin and related glucoregulatory hormone secretion following the removal of such a tumor has never been reported.

## Case Presentation

A 60-year-old man had suffered from lightheadedness for at least 2 years. The day of admission to the hospital, he was found lying in the supine position on the floor with no response to verbal stimulation. The patient's past medical history was unremarkable: no chronic disease, medications, or significant family history were noted. His weight was 65 kg and body mass index (BMI) was 22.2.

### Diagnostic Assessment

**Figure 1.**
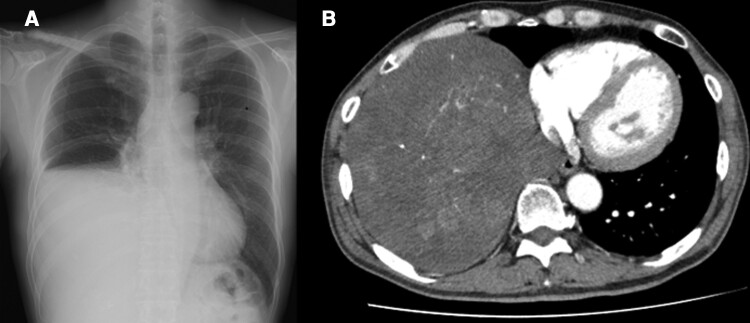
Chest A, x-ray and B, computed tomography (CT) scan. A, Chest radiography displayed a large thoracic tumor measuring 150 × 170 × 120 mm in the right thorax. B, CT scan images showed that tumor was non-homogeneously enhanced by the contrast material.

**Figure 2.**
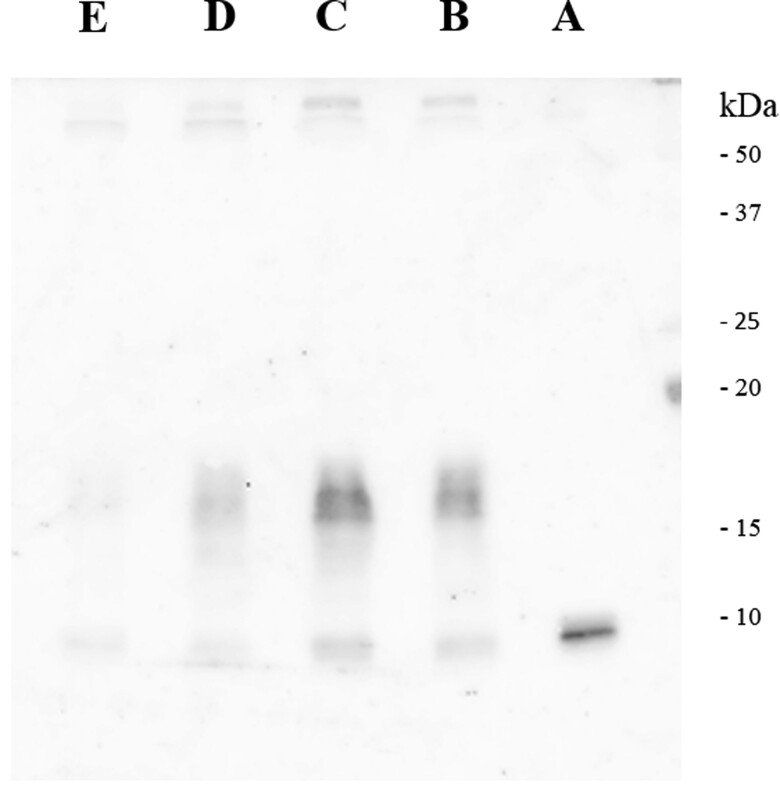
Serum immunoelectrophoresis showing small MWIGF-II (lane A) or high-molecular-weight insulin-like growth factor II (HMW-IGF-II) (lanes B-E). Lane A, the control serum showing standard, small MW, IGF-II (MW: 7.5 kDa) and lanes B to E, patient's serum; B, sample before the operation (before breakfast); C, sample before operation (2 hours after breakfast); sample obtained immediately after surgery; and E, sample obtained 1 day after surgery. The arrow indicates the location of HMW-IGF-II.

On arrival at the emergency department, the patient displayed almost clear consciousness, scoring E4, V4, and M6 on each parameter of the Glasgow Coma Scale with no contributory physical symptoms. Plasma glucose (PG) was measured at 2.1 mmol/L (37 mg/dL) while serum immunoreactive insulin was undetectable (IRI, Abbott, Architect, the lowest detectable limit was 8.3 pmol/L [1.2 mcU/mL]). Chest x-ray showed a large, thoracic tumor (size: 15 × 17 × 12 cm) (Fig. 1A). Contrast-enhanced computed tomography (CT) scan revealed that the tumor was nonhomogeneously enhanced by the contrast material (Fig. 1B). No deficiency of insulin counterregulatory hormones—serum thyrotropin (1.12 mIU/L [1.12 pg/mL]); electrochemiluminescence immunoassay ([ECLIA], Roche Diagnostics K.K.); free thyroxine (16.60 pmol/L [1.29 ng/dL]; ECLIA, Roche Diagnostics K.K.); plasma cortisol (292.4 nmol/L [10.6 μg/dL]; CLIA, Abbott Japan LLC); or adrenocorticotropin (4.53 pmol/L [20.6 pg/mL]; ECLIA, Roche Diagnostics K.K.)—was observed. Western blot analysis of the serum revealed the presence of HMW-IGF-II, which did not change before or after breakfast (Fig. 2). The histopathologic study showed fibrous stroma rich in small blood vessels and a dense arrangement of spindle cells. They were positive for CD34, bcl-2, and CD99 and negative for AE1/AE3. The final diagnosis is a solitary fibrous tumor of the pleura.

### Treatment

Oral administration of up to 3 mg/day dexamethasone was needed to prevent nocturnal hypoglycemia. Surgery was performed for total tumor resection to treat hypoglycemia based on the diagnosis that hypoglycemia resulted from high serum HMW-IGF-II levels from the thoracic neoplasm.

### Outcome and Follow-up

After removal of the benign pleural solitary fibrous tumor, the patient no longer experienced hypoglycemia; serum HMW-IGF-II also disappeared. Glucose metabolism and endocrine tests were carried out postoperative days 5, 202, 272, and 990: a 75-g OGTT on day 272 and other days at fasting. Physical examination showed stable health with proper eating, and the patient gained 6.2 kg by the end of the observation period. His BMI of 23.9 on postoperative day 990 was deemed excellent. Endocrine testing was performed by comparing to a reference group consisting of 46 age- and sex-matched controls selected from the population used in our previous study [2]. The interquartile range from the previous report [2] was adopted as the reference range. The patient data on day 272 were compared with the reference range. Glucagon-like peptide-1 levels (GLP-1, enzyme-linked immunosorbent assay, Cosmic Corp) in healthy Japanese individuals from the 75-g OGTT were used as the reference values (mean ± SE) for GLP-1 [3].

The patient's glycated hemoglobin A_1c_ (HbA_1c_) level increased from 37 mmol/mol (5.5%, preoperative) and stabilized at 41 mmol/mol (5.9%, day 272) at the end of the observation period. Fasting PG (FPG) was 5.3 mmol/L (96 mg/dL) and 2-hour PG at 75 g OGTT was 6.9 mmol/L (125 mg/dL). Homeostasis Model Assessment 2 of β-cell (HOMA2-%B; https://www.dtu.ox.ac.uk/homacalculator/) value was 80.1 (reference range, 64.9-103.7). Basal insulin secretion was within the reference range as indexed by HOMA2-%B. However, glucose-induced insulin secretion was unequivocally depressed: IRI 30 minutes/PG 30 minutes (19.1; reference range, 25.7-57.1) and insulinogenic index (δIRI0-30)/(δPG0-30) (36.4; reference range, 54.5-118.6). The Stumvoll first phase index, the OGTT-derived indices of acute glucose-stimulated insulin secretion (GSIS) [4], were also depressed to 541.5 (reference range, 569.0-1019.0). Levels of basal plasma active GLP-1 were undetectable (< 2.00 pmol/L). The glucose-stimulated level of the GLP-1 after 2 hours of 75-g glucose load was significantly suppressed to 2.23 pmol/L (reference value 9.4 ± 1.7). These data were obtained with normal insulin sensitivity, as shown by the normal Homeostasis Model Assessment 2 of Insulin Resistance (HOMA2-IR) (0.95; reference range, 0.73-1.80).

## Discussion

As far as we know, this case report is the first long-term observation of a patient with NICTH due to an HMW-IGF-II–producing tumor after successful tumor resection. Despite the occasional hypoglycemia before surgery, the patient’s HbA_1c_ was at the upper end of the normal range, 37 mmol/mol (5.5%), so it was considered that his ambient level of PG had been slightly higher than normal [5] even before surgery. Glucose metabolism clearly worsened to the prediabetes stage after surgery, as indicated by an HbA_1c_ of 41 mmol/mol (5.9%) and by fasting PG 5.3 mmol/L (96 mg/dL), 1-hour post-OGTT PG 9.8 mmol/L (176 mg/dL), and 2-h post-OGTT PG 6.9 mmol/L (125 mg/dL). NICTH due to abnormal IGF-II–producing tumor is often left unrecognized.

We performed an extensive endocrine workup of this patient postoperatively because such a study has not been carried out previously. First, glucose metabolism was slightly abnormal and glucose-stimulated acute- or early-phase insulin secretion was impaired, as indicated by the Stumvoll indices, insulinogenic index. GLP-1 secretion was also substantially reduced. On the other hand, basal insulin secretion, measured by HOMA-2%B, was maintained within the normal range, and the degree of insulin resistance, measured by HOMA-2IR, was also within the normal range.

Based on these results, we considered that characteristics of the patient’s abnormalities in insulin-glucose interplay are qualitatively and quantitatively different from what has been observed in historical individuals with a similar level of FPG (96 mg/dL) or the control individuals we employed. Intermittent, yet long-term, hypoglycemia in combination with heightened IGF-II signaling may preferentially damage acute GSIS while preserving basal insulin secretion. This is in contrast to previous reports in patients with insulinoma after tumor removal in whom persistent dysglycemia is rarely seen [6], and the primary residual postoperative abnormality observed is insulin resistance. IGF-II signaling in the pancreatic β cells has generally been regarded as antidiabetic when its strength and timing are appropriate [7, 8]. We hypothesize that impairment of the GSIS after the removal of HMW-IGF-II is an example of “too much of a good thing, ie, prolonged, supernormal IGF-II stimulation of the β cells, can cause irreversible damage to them.” A direct, positive influence of IGF-II on the β-cell–protective GLP-1 action has also been reported in experiments conducted in vitro [8]. If such signaling is operating in the human islet, too much of it might have been deleterious to the islet function, leading to impaired GSIS. Nevertheless, selective impairment of GSIS is a well-known feature of patients with early-phase type 2 diabetes [9] and the evolution of type 2 diabetes in our middle-aged patient might not specifically relate to past IGF-II signaling. The possible stages responsible for impaired glucose responsiveness in β cells might be glucose transport through the membrane, glucose metabolism, ionic and nonionic responses to nutrient signals, and the final membrane fusion [10]. Attenuated GLP-1 response to glucose, another feature of type 2 diabetes, may have contributed to the abnormal insulin secretion in this patient.

In conclusion, we report, for the first time, the glucose metabolism of a patient with NICTH following removal of the HMW-IGF-II–producing tumor. Nine months after the operation, the patient displayed nondiabetic hyperglycemia with impaired GSIS and normal basal insulin secretion. Nonetheless, without head-to-head comparison of results of preoperative and postoperative tests, the conclusion remains speculative. Further studies are required to fully characterize the clinical/endocrine picture of this syndrome.

## Learning Points

- IGF-II–producing tumors are often unrecognized because of their relatively silent clinical presentation.
- The removal of HMW-IGF-II–producing tumor results in successful mitigation of hypoglycemia, although persistent impairment of GSIS with retained basal insulin secretion may be the postoperative abnormality.
- Clinicians should be aware of the patient's glycemic profile, even after successful resection of HMW-IGF-II–producing tumors.

## Data Availability

Some or all data sets generated during and/or analyzed during the present study are not publicly available but are available from the corresponding author on reasonable request.

## Abbreviations

BMI: body mass index
ECLIA: electrochemiluminescence immunoassay
FPG: fasting PG
GLP-1: glucagon-like peptide-1
GSIS: glucose-stimulated insulin secretion
HbA_1c_: glycated hemoglobin A_1c_
HMW: high-molecular-weight
HOMA2-%B: Homeostasis Model Assessment 2 of β-cell
HOMA2-IR: Homeostasis Model Assessment 2 of Insulin Resistance
IGF-II: insulin-like growth factor II
IRI: immunoreactive insulin
NICTH: non–islet cell tumor hypoglycemia
OGTT: oral glucose tolerance test
PG: plasma glucose
